# B Corps: A Socioeconomic Approach for the COVID-19 Post-crisis

**DOI:** 10.3389/fpsyg.2020.01867

**Published:** 2020-07-28

**Authors:** José Manuel Saiz-Álvarez, Alejandro Vega-Muñoz, Ángel Acevedo-Duque, Dante Castillo

**Affiliations:** ^1^Universidad Católica de Santiago de Guayaquil, Guayaquil, Ecuador; ^2^Universidad Autónoma de Manizales, Manizales, Colombia; ^3^Universidad Autónoma de Chile, Santiago, Chile; ^4^Universidad de Santiago de Chile, Santiago, Chile

**Keywords:** social enterprise, social purpose, community-centered, B corporations, Latam

## Abstract

The current global health and economic crisis caused by COVID-19 has opened the possibility to adopt the B Corp model and focus more on the person. Based on grounded theory, we have examined 147 organizations from 14 countries listed at the B Corp Directory for Latin America. Latin American B Corps have traits linked to family-related issues that are distinct from other B Corps located in different continents. Our main findings are that B Corps develop a more inclusive and sustainable economy to benefit society, go beyond the notion of CSR, and move away from traditional companies, as B Corps combine social development and economic growth.

## Introduction

The current global economic crisis caused by the SARS-CoV-2, or COVID-19, is leading toward the possibility of adapting the economy to make it more focused on the person, aligned with the general idea of the B (or Benefit) Corporations (B Corps). The B Corps are hybrid companies defined for-profit. Still, they are socially responsible beyond corporate social responsibility (CSR), as they make profits but not at the expense of stakeholders (Romi et al., 2018). B Corps use an alternative model of business to link for-profit and not-for-profit models (Castellani et al., 2016). They have incorporated a clear societal purpose into their missions, intending to achieve a positive social impact (Perkins, 2019), as they internalize their social and environmental effects (Stubbs, 2017).

The existing economic literature on the B Corps is very scarce. Studies on B Corps have so far focused on solving business organization problems (Hiller, 2013; Rawhouser et al., 2015; Wilburn and Wilburn, 2015; Stubbs, 2017; Bianchi et al., 2020), studying consumers’ perceptions on the B Corps (Marquis et al., 2011; Jin, 2018), and analyzing the combination of economic, social, and ecological objectives to go beyond a CSR defined by purely economic factors (Vargas-Balaguer, 2014; Vargas-Balaguer and Caillet, 2016). However, the triple combination of economic, social, and ecological elements of the B Corps in Latin America has not been studied in detail, which constitutes our primary contribution to our work.

B Corps provide a shared collective identity for internal and external validation; they are focused on societal impact rather than maximizing profits, and they attempt to legitimate this form of sustainable entrepreneurship by influencing the business community and government officials (Stubbs, 2016). As a result, there is a strong public–private collaboration given the externalities generated during the establishment and operation of this type of company.

The public administration can issue B Corp’s certifications in exchange for drawing up Annual reports, or firms can obtain private certificates (Benefit Impact Assessment, BIA) issued by B labs. These B labs measure firm’s externalities on purpose (pc01), certification areas (pc02), stakeholder groups (pc03), and social contribution (pc04), as will be shown in our empirical model. Externalities can also be certified (and re-certified every 2 years) by applying third-party independent standards (Alcorn and Alcorn, 2012; Hiller, 2013; Castellani et al., 2016; Nicholas and Sacco, 2017).

B Corps aim to be certified to attract consumers, as certified B Corps are defined by having a strong social/environmental responsibility, as they are agents of change and customers’ care (Bianchi et al., 2020). Audited and certified B Corps are a third-party signal of achieving a social purpose business model innovation to help organizations to capture value above economic gains. As a result, B Corps participate in activities endowed with ethical, sustainable, or moral goals guided by five B Corp’s certification paths: brand wagoner, reprioritizer, evangelist, inertial benchmarker, and reconfigurer (Moroz and Gamble, 2020).

Regarding the methodology, our study is based on grounded theory and its methods of systematization and knowledge emergence (Glaser and Strauss, 1967; Strauss and Corbin, 1990; Glaser, 1992). Grounded theory, in contrast to the approach obtained by logico-deductive methods, is theory grounded in data that has been systematically collected through social research (Goulding, 2002). We have chosen grounded theory “to conceptualize what is going on in people’s lives—from their perspectives—and to propose theories that can explain and predict processes” (Nathaniel et al., 2019, p. 17). As a result, our research question deals with knowing if the B Corps satisfy social demands or whether they prefer to maximize their profit in a competitive market.

Therefore, the objective of this paper is to investigate the main motivations that lead consumers to buy products and services offered by the B Corps in Latin America (Marquis and Matthew, 2015; B Lab, 2017; Winkler et al., 2019) and how this relationship creates (or not) positive impacts in the community (Cao et al., 2017).

## Background

B Corps are a different type of companies, based on the common good (Groppa and Sluga, 2015), that operate under a model created in 2007 by the American NGO (non-governmental organization) B Lab, which developed a certification to be included under the company’s logo and name. Established in several countries, System B is the organization in Latin America that coordinates B Corps and oversees by organizing corporates’ communication and visibility strategies, as well as implementing training policies to expand these businesses. B Corps show steady growth from their creation (B Lab, 2017). We analyze B Corps for Latin America only, given its heterogeneity and diversity, because Latin American B Corps have traits linked to family-related issues that are distinct from other B Corps located in different continents (Table 1).

**TABLE 1:** B Corps’ characteristics in Latin America vs. the rest of the world.

	Latin America	Rest of the world
A tendency to cooperate internationally	No	Yes
Learning communities	Yes	Yes
Co-creation of solutions with customers only	Yes	Extended
Powerful interest groups	No	Yes
A strong relationship with family firms	Yes	No
Primarily SMEs and social entrepreneurs	Yes	No

*Adapted from Jouny-Rivier and Jouny (2015), Stubbs (2017), and Zebryte and Jorquera (2017).*

B Corps have points in common with the Economy of Communion (Caravaggio, 2018). Both fight against social and/or environmental problems, trade products, and services with the common good in mind and organize critical labor and ecological practices to benefit firms (Cea-Valencia et al., 2016). The origin of the Economy of Communion lies in the implementation of Christian values in the organization (Linard, 2003) by targeting the common good to achieve humanistic management of the firm (Frémeaux and Michelson, 2017). As a result, organizations work for the common good through a lucrative economic activity (Bianchi et al., 2020) but endowed of an open-minded social to benefit society. As a result, the creation of a three-fold social, economic, and ecological benefit transforms organizations into B Corps guided by high standards of transparency and accountability (Vega-Muñoz et al., 2018).

Besides, B Corps perform as public-owned firms (Sharma et al., 2018), because they focus on reducing socioeconomic and environmental distortions (Gueneau, 2015). Consequently, to be classified as a B Corp, organizations must meet high social, environmental, and transparency standards (Hsu and Chen, 2020). In turn, they must commit a shared decision-making procedure by considering the long-term goal of the group (Sanchis-Palacio and Campos-Climent, 2019), as B Corps guided by product newness, low competition, recent technology, and export orientation are more prone of achieving entrepreneurial growth (Carreón and Saiz-Alvarez, 2019).

B Corps work for the common good to benefit society, and the firm (Groppa and Sluga, 2015), where the economic, social, and ecological benefits generated are maximized (Bonilla-García and López-Suárez, 2016). Among these types of companies, the certified B Corps stand out. These accredited organizations are companies that have accepted voluntary third-party social participation and environmental audits conducted by B Lab, a non-profit company (Moroz et al., 2018) focused on suggesting ideas rooted in business opportunities and social work (Xin, 2005; De Smet et al., 2019). Consequently, B Corps must go through a certification process defined in four areas: environment, workers, communities, customers, and the business model (Castellani et al., 2016), and the organization is classified as a B Corp when it obtains a minimum of 80 points out of 200 (Zebryte and Jorquera, 2017). After certification, B Corps are encouraged for continuous improvement to achieving leading positions in their sectors.

Globally, this certification process involves more than 500 national and transnational NGOs (Moroz et al., 2018). External auditors measure and evaluate NGOs’ activities and impacts to analyze to what extent and how audited companies incorporate socially responsible business practices in their operations. Overcoming the certification process involves new business opportunities and better access to resources (Reiser, 2012; Rawhouser et al., 2015). Certified B Corps reach a strong business reputation.

As new training and networking schemes are being designed continuously, certified B Corps improve their score every year (Moroz et al., 2018). They are in a continuous improvement process to increase job creation and the civic commitment of the firm (Masson, 2011). This organizational process creates positive externalities to benefit society, especially stakeholders. Although certified B Corps are encouraged to improve their positive impact on society and the environment endlessly, there is significant variability in how they do so (Conger et al., 2018). These hybrid organizations certify their behavior of fulfilling positive social and environmental actions, in addition to generating economic profitability to benefit their stakeholders (Abramovay et al., 2013).

Directors and managers of these hybrid companies must balance the rights of their shareholders to receive dividends with the interests of their clients, external collaborators, and workers. As a result, both the B Corp’s value chain and its working environment optimize, and the communities where firms operate achieve a higher standard of living (Della Mea, 2013; Ferraro et al., 2015). Consequently, companies are increasingly committed to continuous, stakeholder-driven change toward the implementation of sustained and socially responsible business practices (Delmas and Toffel, 2008; Shepherd and Patzelt, 2011).

Some studies have found that consumers demonstrate a preference for companies that support social or environmental causes (Girling, 2012) and reward these companies by purchasing their products or services (Jin, 2018). As we know, B Corps are hybrid companies between organizations with social purposes and socially responsible firms that propose solutions to social or environmental problems (Masson, 2012). Among these problems to be solved, stand out the access to quality education and conscious consumption, and how to deal with the issues of garbage reduction, obesity and prison recidivism, access to credit, drinking water, energy, quality food, unemployment, ecosystems’ regeneration, and the valuation of biodiversity (Abramovay et al., 2013). As a result, when social and environmental problems worsen, consumers increasingly prefer to buy products and services from responsible hybrid organizations (Bianchi et al., 2020). B Corps do not only operate with the logic of profit, as they increase both working and the living environments of their firms (Barton et al., 2018; Townsend, 2018). These corporate hybrids combine business attributes endowed with (or without) profit (Battilana and Lee, 2014; Rawhouser et al., 2015) to create a positive social or environmental impact to benefit their stakeholders (Chen and Kelly, 2015; Stubbs, 2017). Added to creating value for investors, workers, the community, and environment, innovative B Corps break traditional management paradigms and empower managers, as B Corps have the legal obligation to look upon other interests apart from their shareholders (Tapia and Zegers, 2014) by following a post-positivist perspective guided by subjective influences (Squires, 2009; Nurjannah et al., 2014).

## Methodology

Many researchers work with grounded theory, as they relive the reality of the phenomenon to be studied (Strauss and Corbin, 1998; Birks and Mills, 2015) and review the direction and framework of their research in real time when both new findings and information emerge (Nübold et al., 2017). Grounded theory uses induction-related procedures that generate an explanatory theory of the phenomenon analyzed (Glaser and Strauss, 1967). In this study, we emphasize a conceptual and theoretical approach based on grounded theory (Boe and Torgersen, 2018), since concepts and data relationships are continuously produced and reexamined to be considered rigorous as scientific research (Strauss and Corbin, 1998). Linked to symbolic interactionism, grounded theory ensures to know what is happening and why in a social group (Strauss and Corbin, 1990) to formulate grounded theories with empirical analysis (Martin and Turner, 1986; Strauss and Corbin, 1990; Andrade-Rhor, 2019) on human behavior and the social world (Kendall, 1999). This theory is especially useful when analyzing different organizations and groups (Glaser, 1992) with relatively unstructured information (text data) and theoretical sampling (Hernández-Sampieri et al., 2014; Ilias et al., 2019).

In this work, and given the heterogeneity and diversity of Latin America as a geographical area (López, 2016; Manzano, 2016; Dini and Stumpo, 2018; Bernasconi et al., 2019; Paolasso, 2020), we have used the B Corp Directory for Latin America to contact CEOs and managers working in B Corps. We have chosen Latin America because its diversity and heterogeneity make the conclusions obtained in this study applicable to other regions and continents of the planet. Based on grounded theory, we collected and analyzed the speeches of 147 B Corps representatives from 14 countries: Argentina, Brazil, Chile, Colombia, Costa Rica, Ecuador, Guatemala, Mexico, Nicaragua, Panama, Paraguay, Peru, Uruguay, and Venezuela, before being codified (Cxxx, Product/Service, Country) by using four pre-codes, as identified in the B Corps: purpose (pc01), stakeholders and interest groups (pc02), social contribution, economic growth, and human development (pc03), and certification (pc04). We complemented these pre-codes with codes (axial coding, acxx) that were interpreted as emerging and relationship functions, such as “it is associated with,” “it is part of,” “it is the cause of,” “it contradicts,” “it is one,” and “it is the property of,” to shape a resulting proposition (rpxx). The results are shown and discussed in the next sections.

## Results

**FIGURE 1:**
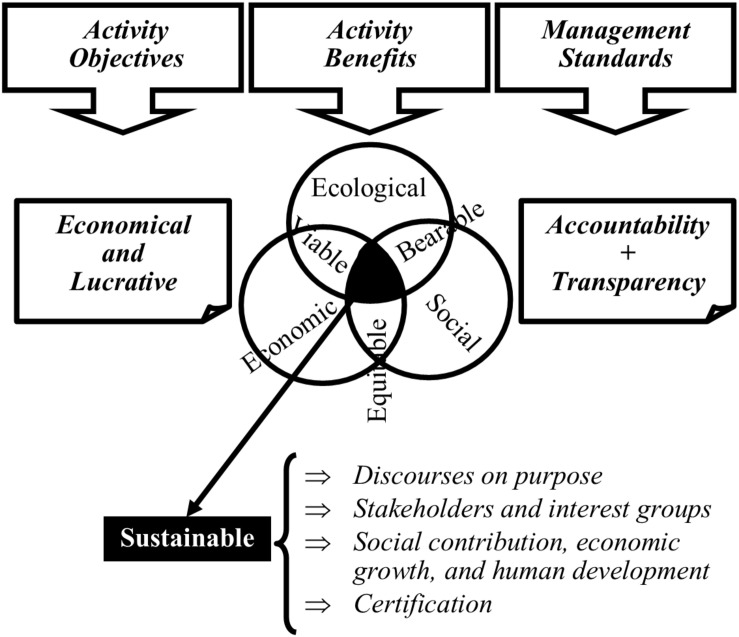
B Corps’ operating scheme.

From the accounts analyzed later, B Corps combine profit (economic, social, and ecological) and social development to create a production model defined by the impact of the firm on four components (or factors): purposes, stakeholders, social contribution, and certification. Business impact is monitored by standards of transparency and accountability in management (Figure 1).

### Discourses on Purpose (pc01)

The B Corps’ Latin American model is moving toward the creation of a commercial and production model for the social, economic, and ecological benefit of stakeholders in contact with the firm. This relationship generates positive externalities, as shown in the following statement from a Brazilian company linked to financial services:

“To mobilize capital for a positive socio-environmental impact by helping different actors in the financial market, such as banks, asset managers, and insurance companies, to incorporate the social, environmental, and government criteria in their decision-making investment process.” (C123, S, BR).

In general, the social and ecological purpose is mostly related to commercial firms, but the most significant economic impact is observed in the production process. This fact is found in the statement made by the following Colombian company:

“The first B Corp came to Colombia in the year 2000 to revolutionize the coffee industry in the country. This new business model has caused producers to receive prices almost three times above the market, but more importantly, it helped to discover exemplary producers who are producing excellent coffee.” (C027, S, CO).

Along with these economic, social, and ecological purposes, the organization includes the perspective of the client to solve the service offered in the market. This goal is achieved with customers’ participation (“learning community”) in the design of the production process (“co-creation of solutions”) and not only with the identification of business problems, as happens in the following Mexican service company:

“We are a B Corp that offers consulting, advisory and training services, and designing processes to co-create solutions tailored to the challenge to be solved in a collaborative environment.” (C140, S, MX).

This triple combination of the economic, social, and ecological purposes in the production stage of the good or service offered to the market must be linked to human resources, as happens at a Peruvian company caring on its workers:

“Our company helps our collaborators, as we respond to their social and environmental needs through a strategic alliance signed with Traperos de Emaús. An organization ruled by the Brothers of Charity.” (C007, S, PE).

From all of the above, the B Corps’ goal is associated with the generation of social, economic, and ecological benefits, only if they are sustainable, viable, bearable, or equitable (*ac01*).

### Stakeholders and Interest Groups (pc02)

When the economic dimension is taken into account only, B Corps’ stakeholders in Latin America tend to privilege interest groups, as they are the primary beneficiaries of the productive aspect of the good (consumer) or service (user) offered in the market. However, when the ecological, social, and economic dimensions of production are combined, interest groups tend to be less powerful. This fact happens in this B Corp of Paraguay, where interest groups benefit from the ecological dimension only, and stakeholders take advantage of the social and economic dimensions of the business model:

***“****Our company aims to offer healthy and inclusive food, to create opportunities in the world of work for people in vulnerable situations, and to solve problems related to the environmental impact generated by waste by recycling waste.” (C017, P, PY).*

Likewise, stakeholders influence the State as explicitly as is recognized in the following statement made by another B Corp in Paraguay:

“We have strengthened the mission of private, social, and government projects. When we created our company, we realized that all the projects with purpose needed computers and reliable communication services.” (C063, S, PY).

B Corps are a model for generating scientific and technological knowledge adjusted to the challenges and dilemmas of contemporary societies. In this case, stakeholders of this Chilean B Corp exceed the margins of being social agents to benefit the community:

“The development of societies is closely linked to their ability to apply scientific knowledge to face the challenges of an increasingly complex and demanding world.” (C041, S, CL).

Thus, in the Latin American B Corps, stakeholders, as part of the company, are also transformed into interest groups (*ac02*).

### Social Contribution, Economic Growth, and Human Development (pc03)

More significant economic development does not necessarily imply higher human development because the factors affecting human development do not equally impact on economic growth. Hence, increasing GDP per capita is not enough. Still, it is necessary to improve living conditions, respect for the environment, and increase social welfare (Amate and Guarnido, 2011). Therefore, the social contribution carried out by B Corps contributes to impelling economic growth and human development, as seen in the following Ecuadorian B Corp:

“Our goal is to solve people’s needs to generate well-being, good health, and quality nutrition, through true solutions by developing the agricultural and livestock sector to generate shared value through good practices and fair trade. We also apply a responsible and innovative approach to the generation of sustainable business and products to promote a healthy planet through productive practices with low environmental impact.” (C049, P, EC).

The same idea is seen in this Chilean B Corp, but now from other areas of social activity:

“The company promotes gender equality within the organization, ensuring equal pay for men and women to promote gender parity through the incorporation of women at the managerial level. This B Corp maximizes the potential of each member of the organization and establishes clear regulations regarding discrimination in the workplace and sexual harassment. Statutes have protected the company’s social mission since its creation. Our firm has been created, first, to do good to the world and, second, to be economically profitable, and not vice versa.” (C042, S, CL).

From all of the above, the social contribution of the B Corps contributes to economic growth and human development in Latin America (*ac03*).

### Certification (pc04)

B Corps are generally interested in getting a certification in any of the three areas (economy, society, and environment) to improve their corporate image, strengthen their brand in the market, and attract new clients and partners. As an example, the following statements from two B Corps in Latin America are included, the first Chilean and the second Colombian:

“Renewable/Clean Energies: Recognizes products/services that reduce GHG emissions by providing renewable or cleaner energy than fossil fuels; Workforce Development: Recognizes the provision of jobs with good quality standards and access to training for people in vulnerable situations.” (C018, P/S, CL).

“Companies face the challenge of remaining relevant in the new digital environment. In this process, they need to minimize their risks, optimize their opportunities, and, above all, fulfill their strategy leveraged on the appropriate technology, Choucair, through focused software testing in business. As a result of this business strategy, we have enabled and enhanced our competitive advantage in the digital transformation of firms.” (C031, S, CO).

Then, B Corps’ certification focuses on achieving a triple balance (social, economic, and ecological) orientation to strengthen the business model of the firm (*ac04*).

In short, the B Corp’s business model in Latin America is oriented toward achieving a triple income statement (economic, social, and ecological) that is beneficial for both the organization and society (rp01).

## Discussion

According to our results, the B Corp model is based on social and economic factors that reduce the centrality of the market, especially when they are combined with ecological and social dimensions. Our idea complements Sharma et al. (2018), who conducted 24 interviews with B Corps’ leaders to verify that B Corps generally change their practice settings while undergoing the evaluation and reevaluation processes to achieve certification. Exogenous factors, such as the firm’s size and sector, and endogenous factors, such as the nature of the firm and their business strategies pursued to maximize their impact on the society, are affected when firms transform into B Corps. B Corps adapt to the market, and they fulfill an inductively derived theoretical framework based on three building blocks: affordability, interpretability, and social references. As a result, organizations change and have a positive impact on administrations (public and private), as they can make good practices happen.

B Corps’ components are not new, as they are globally recognized actions or efforts inserted into the cooperative model and the social and solidarity economy (Campos, 2016; Saiz-Álvarez, 2016; Sanchis-Palacio and Campos-Climent, 2019). Our contribution is how they combine, articulate, and contextualize. The B Corps model renews the activity of the company and explores how these activities can have external effects that stimulate social well-being beyond the limits of the organization (Stephan et al., 2016). In this way, the creation of shared value (CSV) developed by Porter and Kramer (2011) is crystallized in the search for new capitalism that transcends CSR (Muñoz-Martín, 2013). This fact opens the discussion on new forms of social resignification of business (Bocken et al., 2014; de Bakker et al., 2020).

Based on the discourses related to the business practices carried out by B Corps, the combination of productive management skills, good business reputation, and CSR policies linked to the economic, social, and ecological benefits of the firm contributes to achieving a certificate that strengthens the corporate image of the organization. Certification related to corporate identity, rather than a document that classifies the type of production and social contribution carried out by the firm. In other words, being a certified B Corp is the recognition and integration into a learning community.

Having in mind this recognition, organizations are struggling to be certified as B Corps, especially in the case of environmental-related certifications. This desire is especially intense in female entrepreneurs (Grimes et al., 2018) and supports the central theoretical argument of our research that responds to the efforts of participating in identity work by strengthening their sense of self-coherence and distinction through an authentication process to benefit B Corps.

Unlike the efforts made by traditional companies to implement business strategies to stimulate corporate policy and management based on transparency and accountability, transparency and accountability at B Corps are transversal and associated with the production process of goods and services. In this way, B Corps are a pilot experience to advance a useful model to meet social goals demanded by civil society in a socioeconomic context defined by climate change, the regular surge of economic crises, and resource depletion. Consequently, social impact is getting increasingly important on a planet characterized by inequality and social imbalances.

## Conclusion and Perspectives

First, the findings provided by our research confirm, more broadly, that these firms use social-based market laws to respond to the economic, social, and environmental problems B Corps face. As a result, B Corps can develop a more inclusive and sustainable economy to benefit society. Our results are consistent with those recently identified for the Taiwan case in Huang et al. (2019).

Second, B Corps go beyond the notion of CSR. While the CSR model is focused on compensating society for part of the damage generated by the organization, or for the company’s desire to benefit the community where the firm locates, the B Corps model contains, as part of its operation, both the economic contribution and the social purpose. This social purpose is a fundamental part of the production structure and not a consequence of a successful company after profit and capital accumulation.

Third, B Corps move away from traditional companies. Traditional firms continue focusing on maximizing their profits without taking care of market imperfections, social cohesion, and equity. We have shown in this paper that the main motivations that drive B Corps are based on the conviction that it is possible to combine the concepts of social development and economic growth.

This study has expanded the scarce existing research on B Corps that are beyond organizational issues (Stubbs, 2017), consumer behavior, market convenience, and responsible consumption (Bianchi et al., 2020). B Corps are now facing an adverse scenario caused by the SARS-CoV-2 (COVID 19) health crisis. They are learning from the adaptations that firms are implementing to maintain business activity in contexts of social distancing, quarantine, and teleworking. Given its economic advantages and social impact, it is foreseeable that there will be a gradual increase in the creation of B Corps, once the economic effects of this pandemic have disappeared. As a result, these socioeconomic-related firms can be one of the main pillars of the COVID-19 post-crisis.

## Data Availability Statement

Publicly available datasets were analyzed in this study. This data can be found here: https://sistemab.org/empresas-b-america-latina/.

## Author Contributions

ÁA-D, AV-M, and JS-Á: conceptualization. AV-M and DC: methodology design. ÁA-D: formal analysis and writing—original draft preparation. DC: validation. AV-M and JS-Á: writing—review and editing. JS-Á: translate and supervision. All authors have read and agreed to the published version of the manuscript.

## Conflict of Interest

The authors declare that the research was conducted in the absence of any commercial or financial relationships that could be construed as a potential conflict of interest.
